# Meta-Analysis of the Aldehyde Dehydrogenases-2 (ALDH2) Glu487Lys Polymorphism and Colorectal Cancer Risk

**DOI:** 10.1371/journal.pone.0088656

**Published:** 2014-02-18

**Authors:** Hua Zhao, Kui-Jie Liu, Zhen-Dong Lei, San-Lin Lei, Yong-Quan Tian

**Affiliations:** 1 School of Public Health, Central South University, Changsha, China; 2 Department of General Surgery, the Second Xiangya Hospital, Central South University, Changsha, China; The University of Hong Kong, Hong Kong

## Abstract

A number of studies have explored the association of the aldehyde dehydrogenases-2 (ALDH2) Glu487Lys polymorphism and risk of colorectal cancer; however, the results are inconsistent. We performed this meta-analysis to clarify this issue using all the available evidence. Relevant studies were retrieved by searching PubMed. Eleven case-control studies were included in the meta-analysis, representing 2909 cases and 4903 controls. The pooled results based on all included studies showed a decreased colorectal cancer risk in the analysis of the GA genotype *vs*. the GG genotype (OR = 0.81, 95%CI = 0.68–0.98, p = 0.03) and in the dominant genetic model analysis (OR = 0.81, 95%CI = 0.67–0.98, p = 0.03). However, there was no statistical difference in the AA *vs*. GG analysis (OR = 0.74, 95%CI = 0.52–1.06,p = 0.11) and the recessive genetic model analysis (OR = 0.86, 95%CI = 0.69–1.07, p = 0.17). Cumulative meta-analysis based on publication time confirmed these findings. Patients with colorectal cancer had a higher frequency of the GG genotype (OR = 1.10, 95% CI = 1.02–1.20, p = 0.02) and a lower frequency of the GA genotype (OR = 0.89, 95%CI = 0.81–0.98, p = 0.02) comparing with control population. Our results suggested that the ALDH2 Glu487Lys polymorphism may be associated with a decreased risk of colorectal cancer.

## Introduction

Colorectal cancer remains one of the most commonly diagnosed cancers worldwide, with more than one million new cancer cases and 600,000 deaths every year [1]. Colorectal cancer is a multistep, multifactorial disease that involves a complex interplay between genetic and environmental factors. Many gene polymorphisms are associated with risk of colorectal cancer risk [2], [3], [4]. Alcohol consumption has been considered as a risk factor for colorectal cancer according to epidemiologic studies [5], [6]. In fact, ethanol and its metabolite acetaldehyde have been classified as Group 1 human carcinogens [7].

Alcohol in humans is oxidized to acetaldehyde, which in turn is oxidized to harmless acetate by aldehyde dehydrogenases [8]. ALDH2 (aldehyde dehydrogenases-2) is the major enzyme for acetaldehyde elimination, and its polymorphisms determine blood acetaldehyde concentrations after alcohol consumption. The Glu487Lys polymorphism (also named Glu504Lys, or rs671, with the Glutamate corresponding to *1 allele, and Lysine corresponding to *2 allele, the exact position of the variant is 457 of NP_001191818.1 and 504 of NP_000681.2) has been the most frequently studied. A single nucleotide polymorphism at codon 487 in the ALDH2 gene leads to the substitution of glutamate (Glu) by lysine (Lys), which is highly prevalent among east Asians [9]. Such a polymorphism (Glu to Lys, or G to A, or *1 to *2) generates an ALDH2 with much lower activity and causes much higher blood levels of acetaldehyde, which may contribute to susceptibility to carcinogenesis [10].

The Glu487Lys polymorphism has been reported to be associated with many types of cancer, such as esophageal cancer [11], head and neck cancer [12], gastric cancer [13]and colorectal cancer [14]. Several case-control studies have been conducted to clarify the association between this polymorphism and risk of colorectal cancer risk [15]–[25]; however, the results are inconsistent. Chiang's study [15] found that the allele frequency of ALDH2 A was significantly higher in colorectal cancer cases; however, Miyasaka's study[16] found that the A/A genotype of ALDH2 might not be a risk factor for colorectal cancer. Yang's study [17] found that the ALDH2 A/A genotype could increase susceptibility to CRC (adjusted OR = 1.86 (95% CI, 1.12–3.09)); however, Yin's study [19] discovered that the ALDH2A/A genotype was related to a statistically significantly decreased risk of colorectal cancer (adjusted OR 0.55, 95% CI  =  0.33–0.93). In view of the uncertain association between ALDH2 Glu487Lys polymorphism and colorectal cancer risk, we sought to obtain more precise information by conducting a meta-analysis including all of the evidence produced to date.

## Materials and Methods

### Search strategy

Eligible articles were retrieved by searching the PubMed bibliographical database (up to September 20, 2013) using the following combination of keywords: (ALDH2 OR aldehyde dehydrogenase 2) AND (colorectal OR colon OR rectum) AND (polymorphism OR polymorphisms OR variants OR variant). In addition, we checked the references in reviews and in the retrieved articles to avoid missing any of the any relevant studies. There was no restriction on language in the search.

### Inclusion and exclusion criteria

For an article to be included in the meta-analysis, it had to provide the following information: 1) the number of colorectal cancer cases and controls; and 2) the number of individuals with Glu/Glu, Glu/Lys and Lys/Lys in both colorectal cancer cases and controls. Those not designed as case-control studies, systemic reviews, and those that provided no controls or no usable data were excluded.

### Data extraction

Two independent reviewers used a predesigned data extraction table to extract the data. Disagreement was resolved by discussion. The following information was extracted from each included article: journal name, first author, year of publication, population and ethnicity, inclusion and exclusion criteria, source of controls, the number of genotypes in colorectal cancer cases and controls, and the results of the studies.

### Statistical analysis

In the control populations, Hardy–Weinberg equilibrium (HWE) was tested. The strength of the association between the ALDH2 Glu487Lys polymorphism and risk of colorectal cancer was assessed by odds ratios (ORs) with the corresponding 95%CI for each study. The OR and its 95% CI in each comparison were assessed for the genotypes: 1) AA versus GG (A was for the minor allele and G was for the major allele); 2) GA versus GG; 3) the dominant genetic model (AA+GA versus GG); and 4) the recessive genetic model (AA versus GA+GG). The genotype frequencies of GG, GA and AA were also calculated. A chi-squared (χ2) test was used to assess heterogeneity across studies, and *I^2^* statistics were calculated to quantify the proportion of the total variation due to heterogeneity. A fixed effect model was used when there was no heterogeneity among the studies. Otherwise, the random effect model was used. Meta-regression analysis was performed to find the source of heterogeneity and subgroup analysis for country (Japan and China) and design type (HCC(hospital based case-control study) and PCC(population based case-control study)) was conducted. Potential publication bias was assessed using a funnel plot, and the degree of asymmetry was tested by Begg's and Egger's tests (P<0.05 was considered a significant publication bias) [26]. Influence analysis was performed by omitting each study to find potential outliers. Two authors performed the statistical analysis independently and obtained the same results. Statistical analysis was conducted using STATA statistical software (version 11; Stata Corporation, College Station,Texas). p values less than 0.05 were considered statistically significant.

## Results

### Literature selection and study characteristics

**Figure 1:**
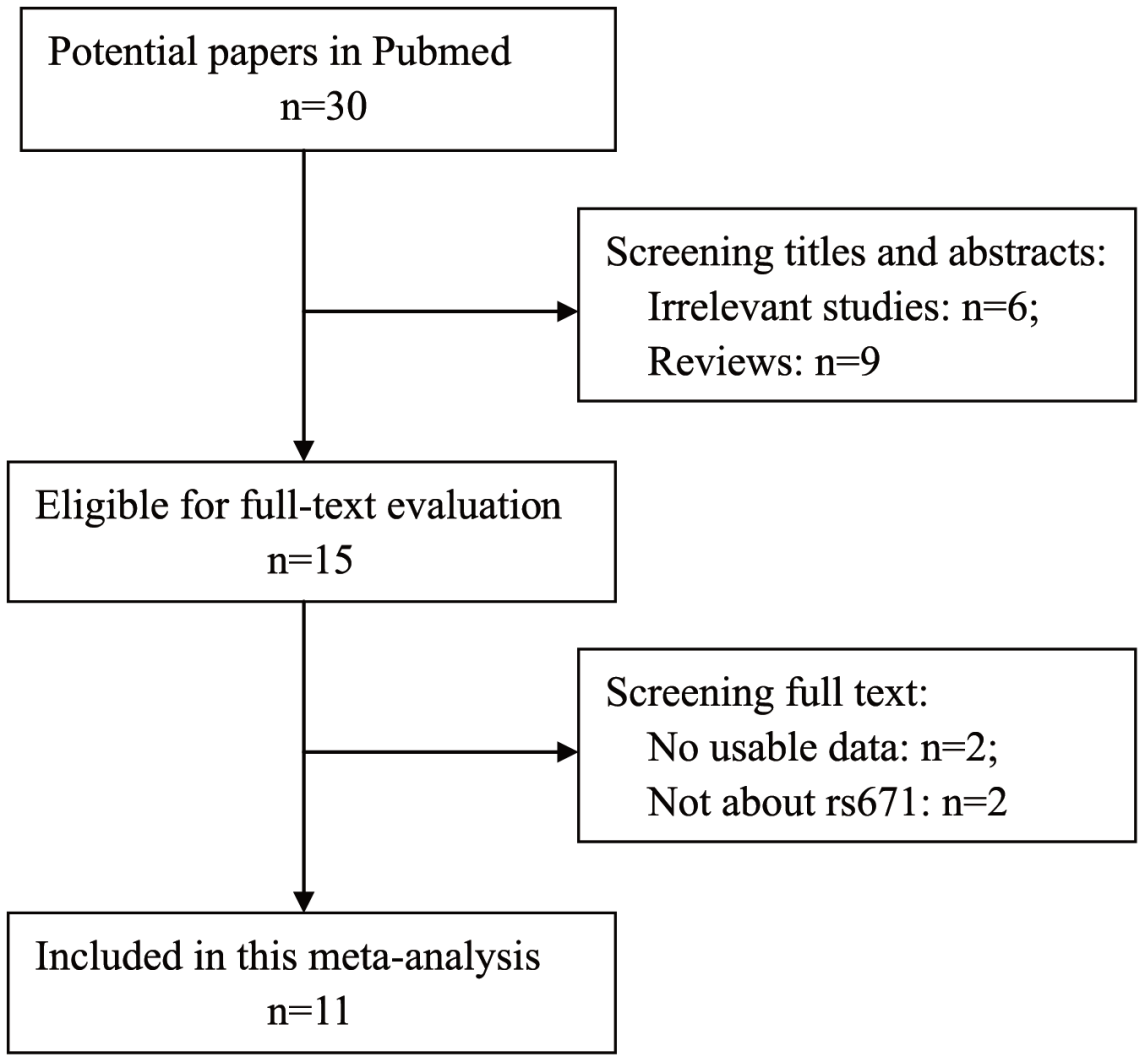
Flowchart of the study selection.

Figure 1 shows the detailed selection procedure. Thirty articles were retrieved from PubMed, fifteen of which were excluded after screening the titles and abstracts (six were irrelevant studies and nine were reviews or meta-analyses). Fifteen relevant articles were selected for detailed assessment by reading the full text. Four of these were excluded (Yin's study [27] and Otani's study [28] had no usable data and Landi's study[29] and Ferrari's study[30] were not about the rs671 polymorphism). Finally, eleven studies met the inclusion criteria (comprising 2909 cases and 4903 controls). Genotype distributions in the controls of Chiang's study[15] and Miyasaka's syudy[16] were not in agreement with the HWE. The detailed characteristics of the studies are shown in Table 1.

**Table 1:** Characteristics of studies included in ALDH2 Glu487Lys polymorphism and colorectal cancer.

Study	Country	Design	HWE	Total	Total	Glu/Glu(GG)		Glu/Lys(GA)		Lys/Lys(AA)	
				cases	controls	Cases	Controls	Cases	Controls	Cases	Controls
Chiang 2012 [15]	China	HCC	No	545	103	304	33	218	53	23	7
Miyasaka 2010 [16]	Japan	PCC	No	48	252	24	112	22	125	2	15
Yang 2009 [17]	China	HCC	Yes	426	785	274	489	119	261	33	35
Gao 2008 [18]	China	PCC	Yes	190	222	131	123	54	90	5	9
Yin 2007 [19]	Japan	PCC	Yes	685	778	400	416	257	309	28	53
Matsuo 2006 [20]	Japan	HCC	Yes	257	768	129	383	104	314	24	71
Otani 2005 [21]	Japan	HCC	Yes	106	224	61	137	36	72	9	15
Kuriki 2005 [22]	Japan	PCC	Yes	72	116	45	64	24	44	3	8
Hirose 2005 [23]	Japan	HCC	Yes	452	1050	299	605	137	390	16	55
Matsuo 2002 [24]	Japan	HCC	Yes	82	118	53	65	26	44	3	9
Yokoyama 1998[25]	Japan	HCC	Yes	46	487	36	443	10	44	0	0
Total number				2909	4903	1756	2870	1007	1746	146	277

### Quantitative data synthesis

**Figure 2:**
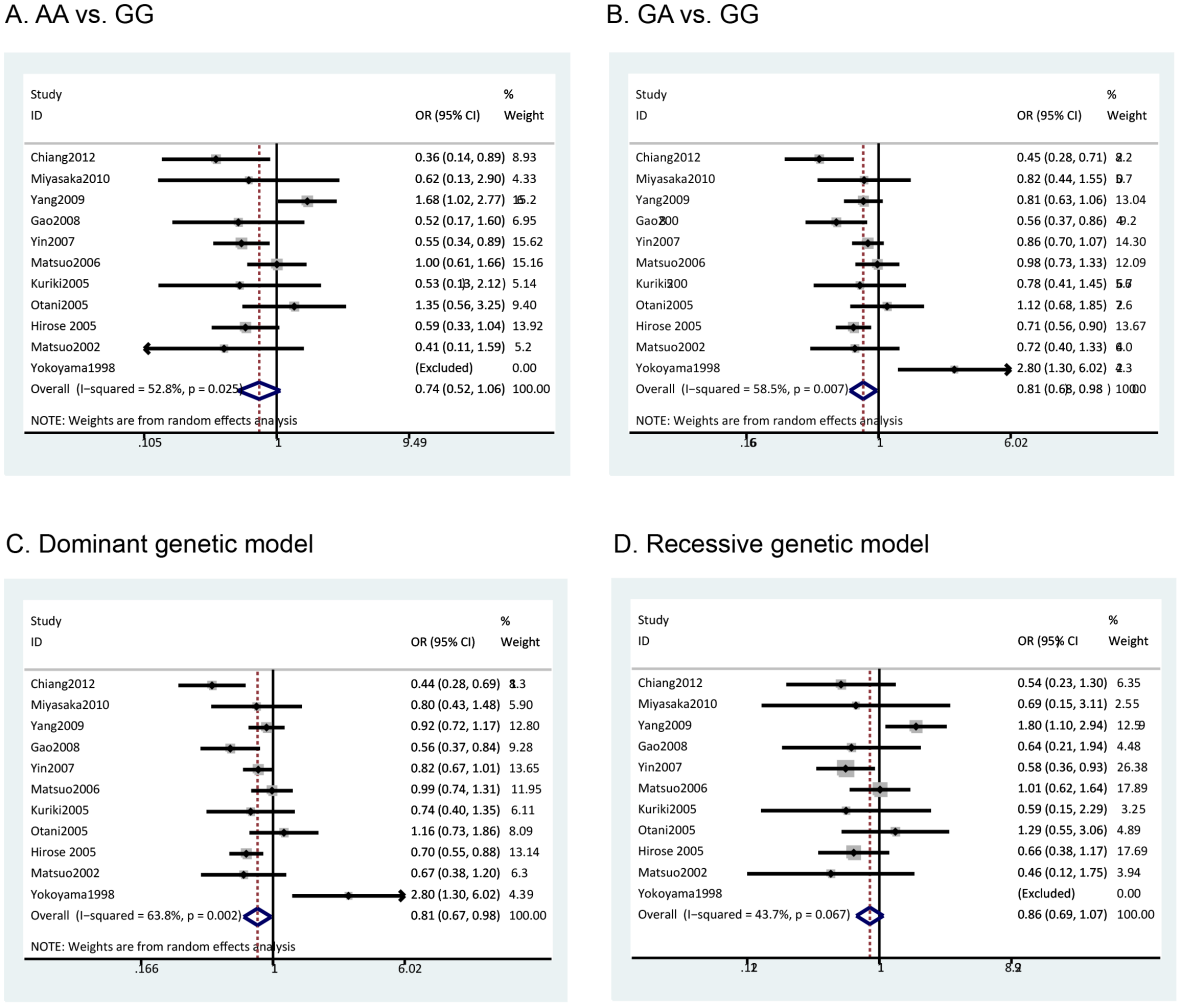
Meta-analysis of ALDH2 Glu487Lys polymorphism and colorectal cancer: A) AA vs. GG analysis; B) GA vs. GG analysis; C) Dominant genetic model analysis; D) Recessive genetic model analysis.

**Figure 3:**
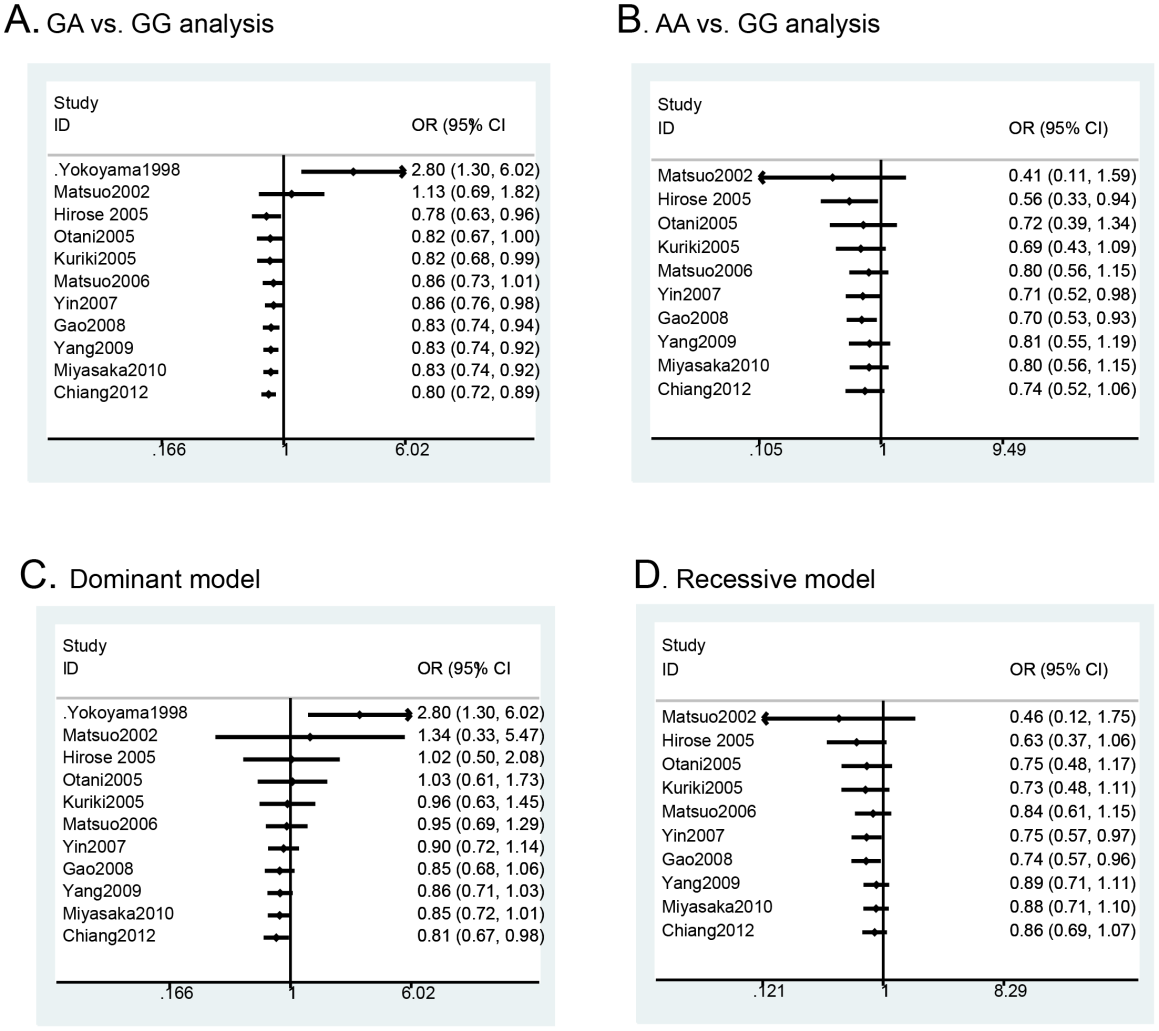
Cumulative meta-analysis of ALDH2 Glu487Lys polymorphism and colorectal cancer: A) GA vs. GG analysis according to publication year; B) Dominant model analysis according to publication year.

The pooled results based on all included studies showed a decreased risk in the analysis of the GA genotype *vs*. GG genotype (OR = 0.81, 95%CI = 0.68–0.98, p = 0.03) (Figure 2B) and in the dominant genetic model analysis (OR = 0.81, 95%CI = 0.67–0.98, p = 0.03) (Figure 2C). However, there was no statistical difference in the analysis of the AA vs. GG genotypes (OR = 0.74, 95%CI = 0.52–1.06, p = 0.11) (Figure 2A) or the recessive genetic model analysis (OR = 0.86, 95%CI = 0.69–1.07, p = 0.17) (Figure 2D). Cumulative meta-analysis based on publication time further confirmed these findings (Figure 3). Furthermore, we calculated the genotype frequencies of GG, GA and AA based on-all included studies, and the results showed that patients with colorectal cancer had a higher frequency of the GG genotype (OR = 1.10, 95% CI = 1.02–1.20, p = 0.02) (Figure S1.A) and a lower frequency of the GA genotype (OR = 0.89, 95%CI = 0.81–0.98, p = 0.02) (Figure S1.B) comparing with the control population. However, there was no significant difference for the AA genotype (OR = 0.87, 95% CI = 0.70–1.08, p = 0.20) (Figure S1.C).

### Tests of heterogeneity and subgroup analysis

We have found heterogeneities in three types of analysis: AA *vs*. GG analysis (χ2 = 19.07, p = 0.03); GA vs. GG analysis (χ2 = 24.10, p = 0.01); and Dominant genetic model analysis (χ2 = 27.61, p<0.01). A random effects model was adopted in these analyses. Meta-regression analysis was performed to find the potential sources of heterogeneity. Unfortunately, the publication year, country, study design type and total sample size were not the significant sources of heterogeneity.

However, we still performed subgroup analysis based on country andstudy design type (HCC, hospital-based case-control study; PCC, population-based case-control study) because such subgroup analysis was valuable. When stratifying the studies by country, we found a decreased colorectal cancer risk in AA *vs*. GG analysis in the Japanese population (OR = 0.71, 95%CI = 0.54–0.93, p = 0.01), a decreased risk in GA *vs*. GG analysis in both the Japanese population (OR = 0.76, 95%CI = 0.76–0.98, p = 0.02) and the Chinese population (OR = 0.67, 95%CI = 0.55–0.82, p<0.01), a decreased risk in the dominant model analysis in the Chinese population (OR = 0.63, 95%CI = 0.39–0.99, p = 0.05) and a decreased risk in the recessive model analysis in the Japanese population (OR = 0.74, 95%CI = 0.57–0.97, p = 0.03). When stratifying the studies by study design, we discovered a decreased risk in AA vs. GG analysis in the PCC group (OR = 0.55, 95%CI = 0.37–0.82, p<0.01), a decreased risk in the GA vs. GG analysis in both the HCC (OR = 0.81, 95%CI = 0.71–0.92, p<0.01) and PCC group (OR = 0.79, 95%CI = 0.66–0.94, p<0.01), a decreased risk in dominant model analysis in the PCC group (OR = 0.76, 95%CI = 0.64–0.90, p<0.01) and a decreased risk in the recessive model analysis in the PCC group (OR = 0.60, 95%CI = 0.40–0.89, p = 0.01). The detailed results are shown in Table 2.

**Table 2:** Summary ORs and 95% CIs of ALDH2 Glu487Lys polymorphism and colorectal cancer risk.

Analysis	n	AA vs.GG		GA vs.GG		Dominant model(GA+AA vs.GG)		Recessive model(AA vs.GG+GA)	
		OR(95%CI)	P/P_het_	OR(95%CI)	P/P_het_	OR(95%CI)	P/P_het_	OR(95%CI)	P/P_het_
Overall	11	0.74(0.52–1.06)	0.11/0.03	0.81(0.68–0.98)	0.03/0.01	0.81(0.67–0.98)	0.03/0.01	0.86(0.69–1.07)	0.17/0.07
Country									
Japan	8	0.71(0.54–0.93)	0.01/0.40	0.86(0.76–0.98)	0.02/0.05	0.89(0.72–1.10)	0.28/0.03	0.74(0.57–0.97)	0.03/0.55
China	3	0.72(0.25–2.14)	0.56/0.01	0.67(0.55–0.82)	0.01/0.06	0.63(0.39–0.99)	0.05/0.01	1.24(0.83–1.85)	0.30/0.03
Study design									
HCC	7	0.94(0.72–1.22)	0.64/0.01	0.81(0.71–0.92)	0.01/0.01	0.87(0.66–1.15)	0.34/0.01	1.01(0.78–1.31)	0.93/0.05
PCC	4	0.55(0.37–0.82)	0.01/0.99	0.79(0.66–0.94)	0.01/0.36	0.76(0.64–0.90)	0.44/0.01	0.60(0.40–0.89)	0.01/0.99

However, when we corrected the p values for multiple testing using the Benjamini-Hochberg false discovery rate method in R package (www.r-project.org), some of the results were no longer statistically significant (p = 0.06 for GA vs. GG analysis and dominant model analysis)(detailed in Table S3), therefore, the results from our meta-analysis may be cautious and further studies were called for this issue.

### Sensitivity analysis

**Figure 4:**
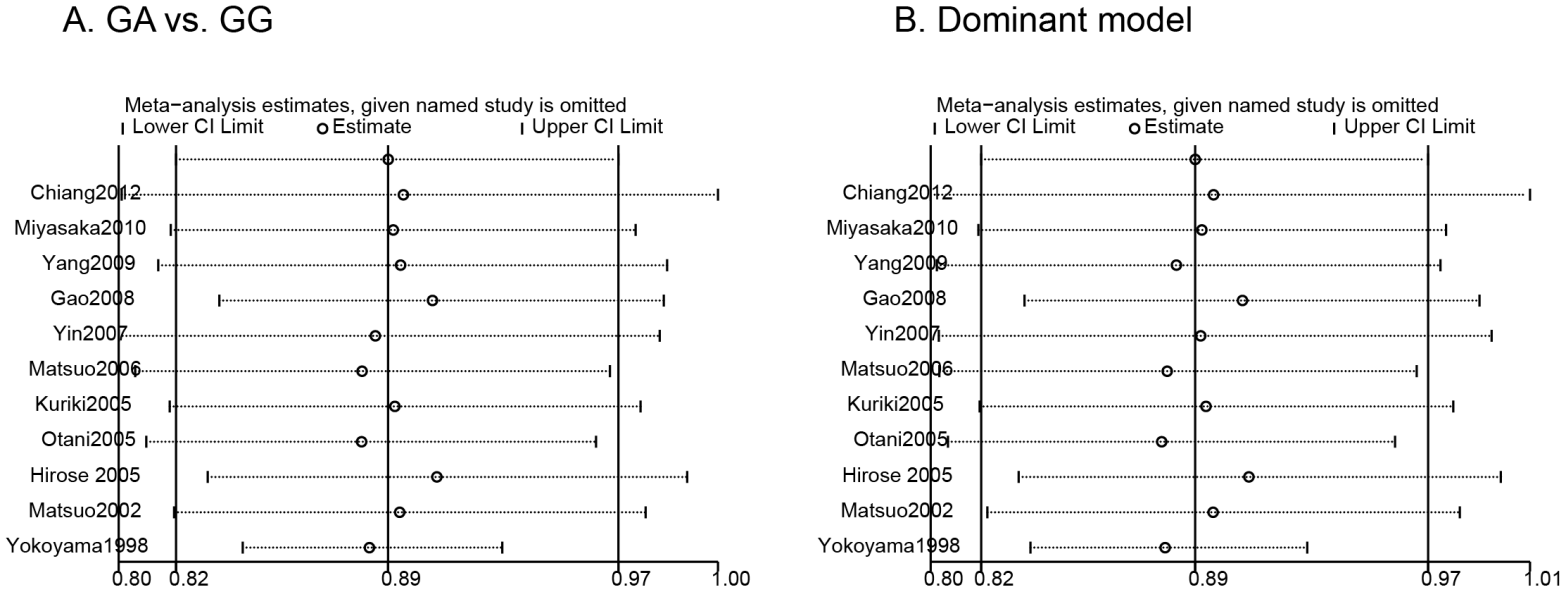
Influence analysis for ALDH2 Glu487Lys polymorphism in the overall analysis: A) GA vs. GG analysis; B) Dominant model analysis.

Influence analysis was conducted to assess the sensitivity of each individual trial on the pooled ORs by sequential omission of each individual trial. The results suggested that no individual trial significantly affected the pooled ORs in the GA vs. GG analysis and dominant model analysis (Figure 4 A and B).

### Publication bias

**Figure 5:**
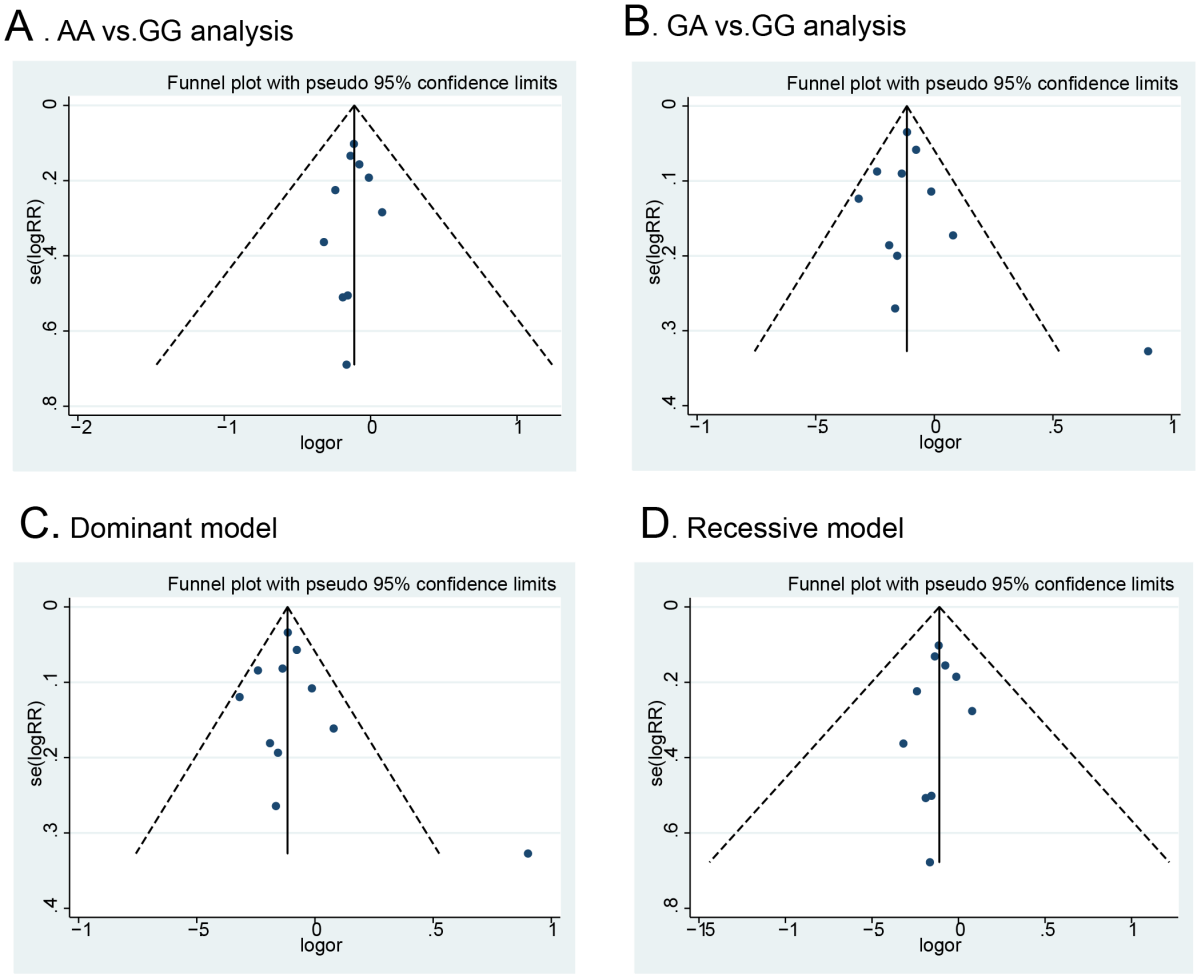
Funnel plot of ALDH2 Glu487Lys polymorphism and colorectal cancer risk for publication bias.

Potential publication bias was examined qualitatively by funnel plots and estimated quantitatively by Begg's and Egger's tests. As shown in Figure 5, the shapes of the funnel plots did not indicate any evidence of obvious asymmetry. Moreover, the p values from Begg's test and Egger's test were all greater than 0.05 (Table S2), indicating no publication bias

## Discussion

To the best of our knowledge, this is the first meta-analysis to evaluate the association between an ALDH2 polymorphism and risk of colorectal cancer. Our meta-analysis included eleven studies with a total of 2909 cases and 4903 controls for the Glu487Lys polymorphism. In this meta-analysis, we discovered a decreased CRC risk in the analysis of the GA genotype *vs*. the GG genotype and in the dominant genetic model analysis. Cumulative meta-analysis further confirmed these findings. Furthermore, we found a higher frequency of the GG genotype and a lower frequency of the GA genotype in CRC patients. These results are interesting and unexpected.

In general, ALDH2 plays a key role in clearing acetaldehyde generated from alcohol consumption; therefore, the acetaldehyde concentrations after drinking are mainly dependent on the enzyme activity of ALDH2 [31]. In ALDH2 GA and ALDH2 AA subjects, the blood acetaldehyde concentrations after drinking alcohol were 6 and 19 folds higher, respectively, than that in ALDH2 GG subjects in case of assuming the same amount of alcohol [32]. The accumulation of acetaldehyde in the blood and repeated high exposure to acetaldehyde after drinking may contribute to the development of colorectal cancer[33]. According to this, the GA and AA genotype should be risk factors for cancer. In fact, a previous study found that GA and AA were associated with an increased risk for esophageal cancer [34].

However, our meta-analysis shows very different results. In our meta-analysis, the GA and AA genotypes may be a protective factor for colorectal cancer risk. It might be because ALDH2 GA and AA subjects can develop intense facial flushing responses with nausea, headache,drowsiness and other unpleasant symptoms resulting from high blood acetaldehyde levels after alcohol consumption[35]. This unpleasant discomfort may prevent people from consuming alcohol and may keep them from developing alcoholism thus they have much lower chance to expose to the carcinogen acetaldehyde [36], which may decrease the risk of developing colorectal cancer. Studies have shown that there were fewer heavy drinkers among people carrying the AA genotype [37]. Therefore, the protective role of the AA genotype may be caused by decreased alcohol consumption. In fact, certain studies have demonstrated a protective relationship of ALDH2 GA genotype with hepatic carcinoma [38] and the ALDH2 AA genotype with esophageal cancer [37], [39] and liver cirrhosis [40].

However, does the protective role of GA and AA genotype for CRC only exist among the non- or rare drinkers or even among heavy drinkes? It is difficult to answer this question. because we could not perform subgroup analysis according to drinkers and non-drinkers to clarify the alcohol-genotype interaction. Further study is needed to explore this important issue.

In our meta-analysis, the overall recessive model analysis and AA vs.GG analysis only showed a tendency of protective role for AA genotype rather than statistical significant. It may be due to the low frequency of AA genotype in the population (The frequency of AA genotype is only 5.65% in the control population included in our meta-analysis, OR = 0.042, 95%CI = 0.037–0.048). In the sub-group analysis, we found a decreased colorectal cancer risk in AA vs. GG analysis and in recessive model analysis in Japanese population but not in Chinese population, it may be due to the A allele in Japanese sample is much higher than that in Chinese sample in HapMap sample [41]. In fact, the frequency of AA genotype is much higher in Japanese people(frequency of AA genotype is 5.96%, OR = 0.045,95%CI = 0.038–0.052) than that in Chinese people(frequency of AA genotype is 4.59%, OR = 0.035,95%CI = 0.026–0.047) in the studies included in this meta-analysis (p = 0.005).

Although the primary results of this meta-analysis are suggestive, some limitations still exist. Firstly, we could not perform subgroup analysis according to drinking status because of the lack of sufficient original data; therefore, our results may be biased because the drinking status may influence the risk of CRC. Secondly, there was heterogeneity between studies of ALDH2 polymorphisms, and and meta-regression analysis was failed to find the potential heterogeneity. Thirdly, all of the studies were conducted in Japan and China, and other high risk areas of CRC did not explore the relationship between ALDH2 polymorphism and CRC. Therefore, further studies are warranted in other high risk areas. Fourthly, although the genotype distributions in the pooled controls from the included studies were in agree with HWE, genotype distributions in the controls from Chiang's study[15] and Miyasaka's study[16] were not in agreement with HWE, therefore, the results may be biased. Lastly, publication bias may have occurred, although the funnel plot did not indicate this; negative findings were likely to be unreported.

In conclusion, this comprehensive meta-analysis has evaluated all published data currently available on the ALDH2 Glu487Lys polymorphism and risk of colorectal cancer. Our meta-analysis suggested that the GA and GA+AA genotypes may reduce the risk of CRC compared with the GG genotype, which may be explained by the unpleasant symptoms of ALDH2 A carriers preventing them from consuming alcohol.

## Supporting Information

Figure S1 **Meta-analysis of ALDH2 Glu487Lys genotypes and colorectal cancer risk: A) GG genotype frequency; B) GA genotype frequency; C) AA genotype frequency.**
(TIF)

Table S1 **PRISMA checklist.**
(DOC)

Table S2 **P vaues in the Egger and Begger's test.**
(XLS)

Table S3 **Summary OR and 95% CI adjusted for multiple testing using BH-FDR method.**
(XLS)
