# Supplementary figures and images for: Meta-Analysis of the Aldehyde Dehydrogenases-2 (ALDH2) Glu487Lys Polymorphism and Colorectal Cancer Risk
Source: PLoS One. 2014 Feb 18;9(2):e88656. doi: 10.1371/journal.pone.0088656 (PMC3928247; doi:10.1371/journal.pone.0088656)

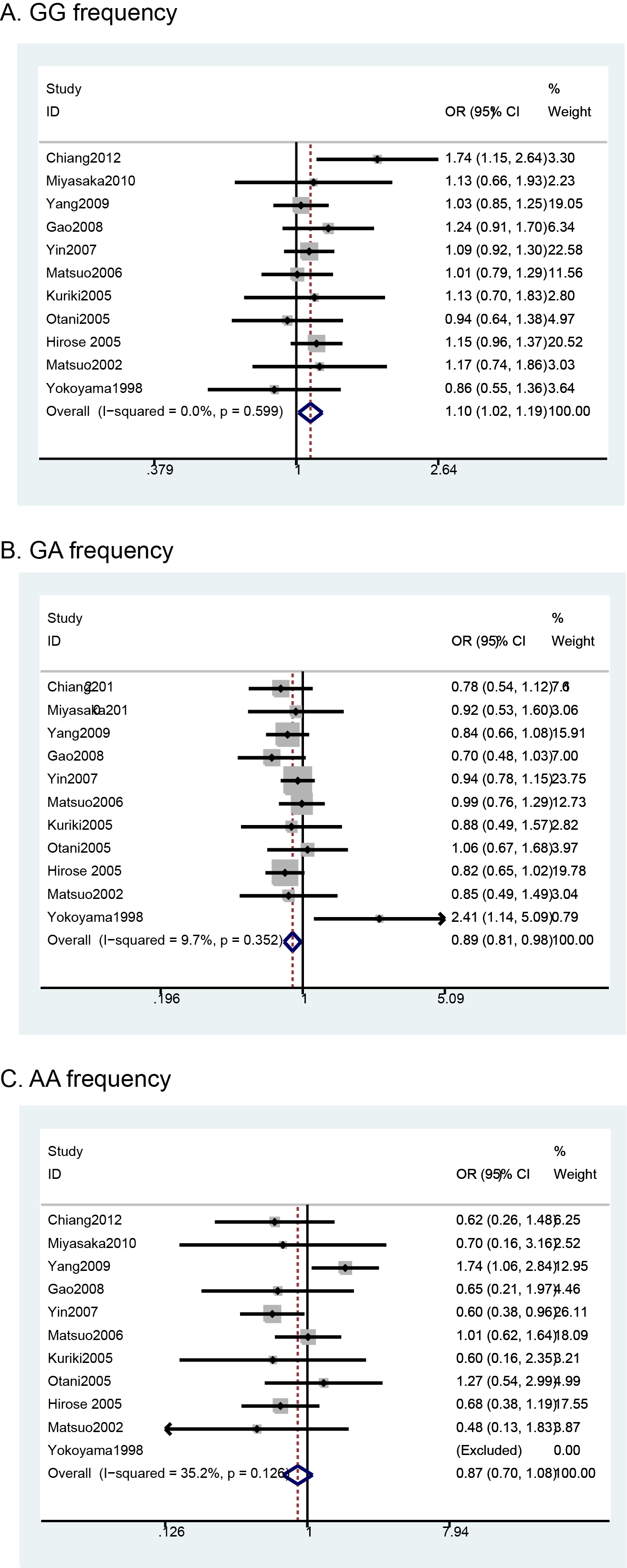

Supplement: Figure S1 — Meta-analysis of ALDH2 Glu487Lys genotypes and colorectal cancer risk: A) GG genotype frequency; B) GA genotype frequency; C) AA genotype frequency. (TIF) [file pone.0088656.s001.tif]
